# Surface Nano-Structuring by Adsorption and Chemical Reactions

**DOI:** 10.3390/ma3094518

**Published:** 2010-08-27

**Authors:** Ken-ichi Tanaka

**Affiliations:** Saitama Institute of Technology, Research Center of Advanced Sciences 1690 Okabe, Fukaya, Saitama, Japan; E-Mail: ktanaka@sit.ac.jp; Tel.: +081-(0)58-585-6874; Fax: +081-(0)58-585-6874.

**Keywords:** self-assembly, nano-structuring, nano-dots and lines, formation and array of quasi-compounds, reaction of surface atoms, phase boundaries, reconstruction, Cu(100), Cu(110), Ag(110), Ni(110), Au(111)-hex, Si(111)-7 × 7, electronic and magnetic properties of nano-metals

## Abstract

Nano-structuring of the surface caused by adsorption of molecules or atoms and by the reaction of surface atoms with adsorbed species is reviewed from a chemistry viewpoint. Self-assembly of adsorbed species is markedly influenced by weak mutual interactions and the local strain of the surface induced by the adsorption. Nano-structuring taking place on the surface is well explained by the notion of a quasi-molecule provided by the reaction of surface atoms with adsorbed species. Self-assembly of quasi-molecules by weak internal bonding provides quasi-compounds on a specific surface. Various nano-structuring phenomena are discussed: (i) self-assembly of adsorbed molecules and atoms; (ii) self-assembly of quasi-compounds; (iii) formation of nano-composite surfaces; (iv) controlled growth of nano-materials on composite surfaces. Nano-structuring processes are not always controlled by energetic feasibility, that is, the formation of nano-composite surface and the growth of nano-particles on surfaces are often controlled by the kinetics. The idea of the “kinetic controlled molding” might be valuable to design nano-materials on surfaces.

## 1. Introduction

It is known that the truncated crystal surface is strained through several layers, and the surface prefers to adopt a less strained structure. Therefore, the adsorption of atoms or molecules, the reactions of surface atoms with adsorbed species, and the array of reaction products are influenced by local strain on the surface. The phenomena influenced by such a surface nature are unpredictable, so that the nature of the surface was figuratively expressed by the saying “God made the bulk and the Devil made the surface”. The development of various surface sensitive tools has removed the veil of the capricious nature of surfaces. In particular, low energy electron diffraction (LEED) has enhanced our knowledge about the ordered structure of the surfaces. In 1964, Germer [1] wrote the following sentence in his review: “this reconstruction of surfaces by adsorption of foreign atoms upon them is without doubt the most significant result that has been obtained up to this time from low energy electron diffraction (LEED) studies”. However, we could say that the process or mechanism of the restructuring of surfaces at the atomic level is still speculative.

In 1983, scanning tunneling microscopy (STM) was presented by Binnig, Rohrer, Gerber, and Weibel [2,3], which enabled us to inspect the real surface with atomic resolution. The reconstruction process of a Ni(110) surface in H_2_ is a good example. Ertl *et al.* [4,5] showed a phase change of a Ni(110) surface depending on the coverage of H at T < 180 °K, a p(2 × 1) Ni(110)-H at θ_H_ = 1.0 and a p(1 × 2) Ni(110)-H at θ_H_ = 1.5, by the LEED. However, Nielsen *et al.* [6] showed a quite different irreversible reconstruction of a Ni(110) surface by the adsorption of H_2_ at room temperature by using STM, that is, Ni atoms released from the terrace make new rows along the [1-1 0] direction. The p(2 × 1)Ni(110)-H surface reconstructed at room temperature is essentially different from the formation of reversible p(2 × 1)Ni(110)-H surface at temperatures below 180 °K. One can now verify the structure on the atomic scale by using STM, but the process still remains speculative, because it is difficult to inspect transient atoms during restructuring. That is, we can detect only stabilized atoms and completed structures. For example, it was so often explained as if adsorbed H atoms react with Ni atoms provided on the surface by thermodynamic equilibration [7]. It is the same for the growth of (-Cu-O-) chains on Cu(110) surface, that is, migrating Cu and O-atoms are trapped at the terminal of (-Cu-O-) chains. However, the rate of growth of (-Cu-O-) on the surface is difficult to explain by Cu atoms ejected by a thermodynamic equilibration. A new concept of “quasi-molecule (pseudo-molecule)” was proposed in 1993 [8,9] to explain the rapid release and rapid migration of metal atoms over the terrace in the presence of O_2_ or H_2_. We supposed the formation of “quasi-molecules” is the driving force to release Ni or Cu atoms from the surface, that is, intermediate species such as (NiH)*, (NiO)*, and (CuO)* are formed by the reaction of surface atoms with adsorbed H or O atoms. Buisset *et al.* [10] reported mobile (-Cu-O-) chains on the Cu(110) surface at 77 °K, which suggests a highly mobile species, which might be the mobility of some composite species [maybe (CuO)*] of the (-Cu-O-) chain. The notion of a quasi-molecule explains well an interesting transportation of Cu atoms from a STM W-tip coated with Cu onto Ag(110) surface in the presence of oxygen as will be discussed below [11]. The quasi-molecule is a hypothetical intermediate species provided by the reaction of surface atoms with adsorpbed species. It should be pointed out that quasi-molecules are stabilized on a specific surface by forming stoichiometric “quasi-compounds” by self-assembly. The presence of (NiH)* and (NiO)* species was confirmed by the results of competitive adsorption of O_2_ and H_2_ on Ni(110) given by Besenbacher *et al.* [6,12]. As discussed in this paper, the idea of quasi-molecules and quasi-compounds is quite valuable to understand various phenomena taking place on the surface. A typical example is the sequential change of p(nx1)Ag(110)-O surface (n = 7, 6, ---, 2) depending on oxygen coverage on Ag(110), which is explainable by a self-organization of a (AgO)* quasi-molecule over the surface [13,14], but is difficult to explain by ordered adsorption of oxygen atoms. A self-assembled array of quasi-compounds occurs by weak interaction, which is different from the growth of stable compounds on the surface. As discussed below, the surface provides a two dimensional reactive space as well as a two dimensional periodic space for self-assembly. Therefore, we can prepare new materials having controlled configurations given by thermodynamics feasibility or kinetic preference.

## 2. Self-Assembly of Adsorbed Molecules and Atoms

**Figure 1:**
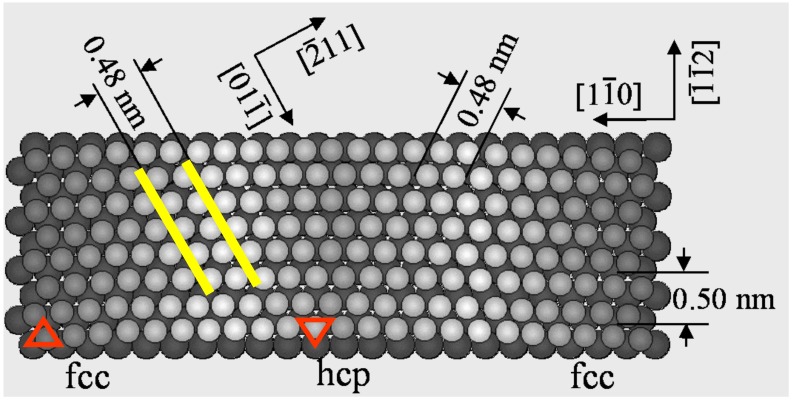
A model of the reconstructed Au(111) surface. About 4.2% of anisotropic shortening of the Au-Au distance occurs along the [1-1 0] direction.

An anisotropic or isotropic crystalline structure influences not only on the array of surface atoms but also the array of adsorbed atoms and molecules. An anisotropic “herring born structure” of Au(111) surface is quite unique, because this surface is formed by anisotropic shortening of the Au-Au distance along the [1-1 0] direction by ca. 4.2%, as shown in Figure 1. A self-assembled array of alkanes or alcohols reflects the attractive or the repulsive weak interaction on the anisotropic surface.

**Figure 2:**
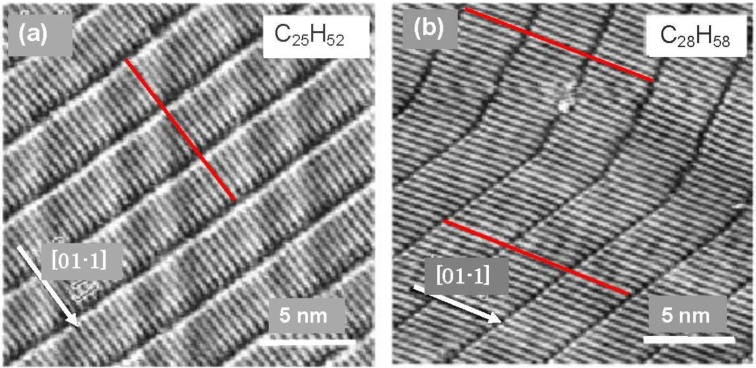
Two dimensional crystallization of *n*-C_25_H_52_ and *n*-C_28_H_58_ by self-assembly on a reconstructed Au(111) surface. The domain boundary is perpendicular in the array of an odd carbon alkane (*n*-C_25_H_52_), whereas it is ca. 120° with respect to the molecular axis in the array of an even carbon alkane (*n*-C_28_H_58_) by sliding the molecules along the axis [16,17].

Uosaki *et al.* [15] reported a two dimensional crystalline molecule made by self-assembly of alkanes by using scanning tunneling microscopy (STM). Systematic studies were carried out by Xie *et al.* [16,17], that clarified the crystallizing mechanism by the anisotropic interaction of *n*-alkane molecules (C_n_H_2n+2_) on a reconstructed Au(111) surface. A similar phenomenon was also shown in aqueous solution by He *et al.* [18]. Although Marchenko *et al.* [19,20] proposed an end-on adsorption model of paraffins (carbon number = 18–26) on a Au(111) surface, it was evidently contradicted by a co-adsorption of *n*-C_17_H_36_ and C_36_H_74_ [16] and a systematic experiment with *n*-C_n_H_2n+2_ (n = 14–38) [17] proved the side-on adsorption of alkanes on reconstructed Au(111) surfaces. About 4.2% anisotropic lattice shortening occurs along the [1-1 0] direction, which results in lattice distances of 0.50 nm along the [−1-1 2] direction and 0.48 nm along the [−2 1 1] or [1-2 1] direction. No adsorption of small molecules is observed on a reconstructed herring-bone Au(111) surface, but rather a well ordered self-assembled adsorption layer is formed by attractive interaction among the long *n*-alkane chains. This reconstructed Au(111) surface has a lattice distance of 0.48 nm toward the [−2 1 1] direction, which is very close to the molecule-molecule distance of crystalline *n*-C_36_H_74_. Therefore, the mutual attractive interaction of *n*-alkane molecules on the herring-bone Au(111) surface is optimized by aligning along the [0 1-1] direction, and the array of *n*-C_25_H_52_ and *n*-C_28_H_58_ molecules occurs as shown in Figure 2(a) and Figure 2(b) [16].

**Figure 3:**
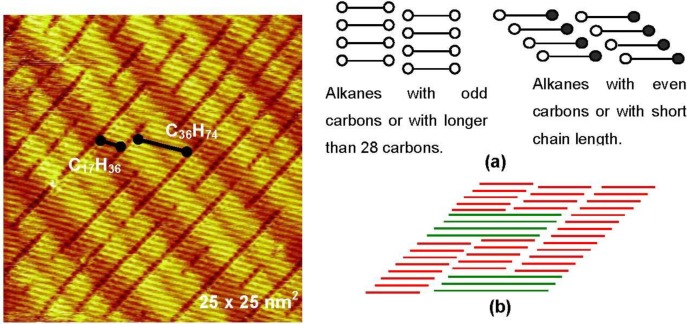
Two dimensional eutectic nano-crystal phase of *n*-C_17_H_36_ and *n*-C_36_H_74_ (70:30) on a reconstructed Au(111) surface formed in a saturated solution of *n*-C_36_H_74_ in *n*-C_17_H_36_ [13]; (**a**) Models of the array of odd and even carbon alkanes by forming either perpendicular or tilted domain boundary to the molecular axis; (**b**) A model of an eutectic phase of two alkanes with twice different chain length.

The contacting part of the alkane chains is maximized when the domain boundary becomes perpendicular to the molecular axis. In this case, however, the repulsive interaction of terminal—CH_3_ groups will be at a maximum. However, the repulsive interaction of -CH_3_ groups will be different when alkane molecules have odd or even numbers of carbon atoms because of their zigzag structure, as illustrated in Figure 3(a). In fact, the angle of the molecular axis to domain boundary depends on both the chain length and the number of odd or even carbon atoms, that is, the rivalry in the attractive interaction of molecular chains and the repulsive interactions of the terminal methyl groups.

Systematic experiments by Xie *et al.* [16,17] showed that the domain boundary becomes right angled to the alkane molecules when the carbon number is odd (*n*-C_25_H_52_ and *n*-C_33_H_68_ [17]). On the other hand, in the case of *n*-C_28_H_58_, the domain boundary lies at about 120 (or 60) degrees with respect to the alkane molecules by a parallel sliding of the alkane molecules in their array, as shown in Figure 2(b).

The domain boundary prefers to be perpendicular to the molecular axis when the alkane molecule is larger than 28 carbons (n > 28), even if the carbon number is even [17], that is, the attractive interaction exceeds the repulsion of the –CH_3_ groups. However, coadsorption of an even number alkane (*n*-C_36_H_74_) and a short odd number alkane (*n*-C_17_H_36_) on the reconstructed Au(111) surface makes an interesting eutectic phase, as shown in Figure 3 [16]. The eutectic nano-crystal phase is composed of *n*-C_17_H_36_ and *n*-C_36_H_74_ having their molecular axes tilted to the domain boundary, that is, the repulsive interaction of -CH_3_ is influential in the eutectic nano-crystal phase.

When a molecule adsorbs weakly on a surface, the surface will be only slightly strained by the adsorption, so that the mutual interaction between adsorbed molecules more effectively influences their self-assembly. On the other hand, when adsorbed atoms or molecules have strong adsorption bonds with the surface atoms, the surface lattice will be strained. In this case, the surface adopts a less strained and lower total energy structure. Accordingly, when the lattice distortion exceeds a critical level, the surface undergoes reconstruction by changing the array of atoms or by ejecting surface atoms. If the strain induced by the adsorption is increased non-linearly as the adsorption increases, the adsorption prefers to make small domains. The adsorption of oxygen on a Cu(100) surface is a good example.

Lee and Farnsworth [21] observed first an unusual “four-spot” LEED pattern at low oxygen coverage on a Cu(100) surface caused by the adsorption of O_2_, which was not the expected c(2 × 2)-O structure. On the other hand, formation of c(2 × 2)-O structure on a Cu(100) surface was reported by Sotto [22] at low temperature (220–350 °K), and the formation of a (2√2 × √2)R45° reconstructed surface at an oxygen coverage of 0.5 was reported by many investigators [23,24], and its structure was well solved. However, the question of the transfer of the four-spot Cu(100)-O surface to the (2√2 × √2)R45°-O surface by increasing oxygen coverage was unsolved for almost 30 years although it was studied using various new tools such as high resolution electron energy loss spectroscopy (HREELS) [25,26], X-ray diffraction (XRD) [27], and scanning tunneling microscopy (STM) [28,29]. Formation of disordered phases and disordered growth of one dimensional (-Cu-O-) chains on the Cu(100) surface was speculated [28]. The results of surface-extended x-ray absorption fine structure (SEXAFS) [30,31], high-resolution electron energy-loss spectroscopy (HREELS) [25,26], and X-ray photoemission spectroscopy [32] suggested a local disordered adsorption of oxygen or the adsorption of oxygen atoms on different sites.

**Figure 4:**
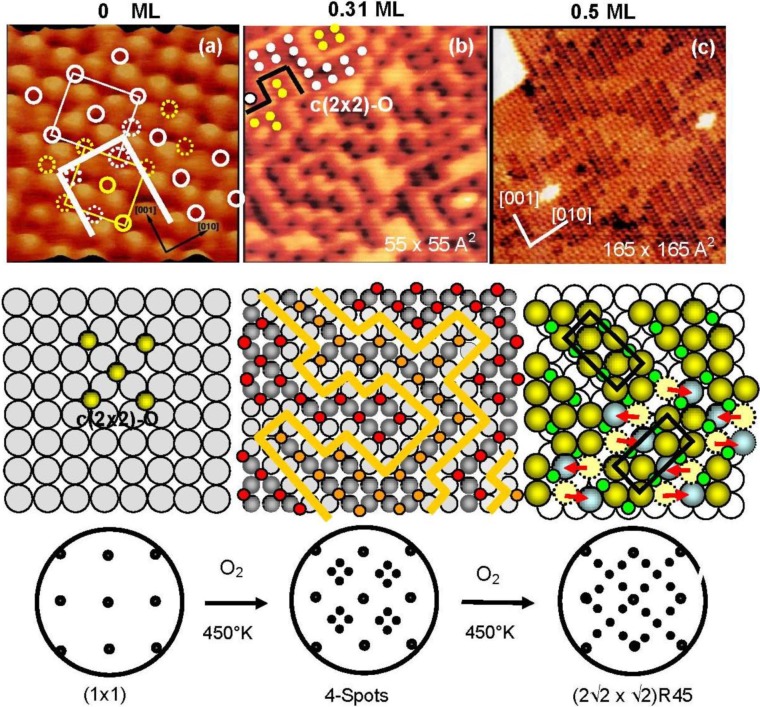
STM images of the Cu(100) surface with different oxygen coverage, (**a**) Clean Cu(100); (**b**) Four spots surface at 0.31 mL oxygen coverage (exposed to O_2_ for ca. 8 L (10^−7^ Torr × 80 s) attained at ca. 450 °K ), and (**c**) (2√2 × √2)R45° surface at ca. 0.5 ML coverage, and their model structures. Cu-atom missing ditches of the (2√2 × √2)R45° surface can change the orientation of a ditch from the [001] to [010] or vice versa by shifting Cu atoms to an equivalent neighbor sites [33,34].

The origin of the four-spot Cu(100)-O surface and its transformation to the (2√2 × √2)R45° structure were finally clarified by our *in-situ* STM studies [33,34]. The STM images of the Cu(100) surfaces with different oxygen coverage are shown in Figure 4. The dark dents observed in the images (a) and (b) are the oxygen atoms adsorbed on the four-fold hollow sites in a small c(2 × 2) domains as illustrated with marks in Figure 4(a). The surface in image (b) shows the four-spot LEED pattern, and the bright zigzag lines of image (b) are the phase boundaries of the nano-c(2 × 2)-O domains.

**Figure 5:**
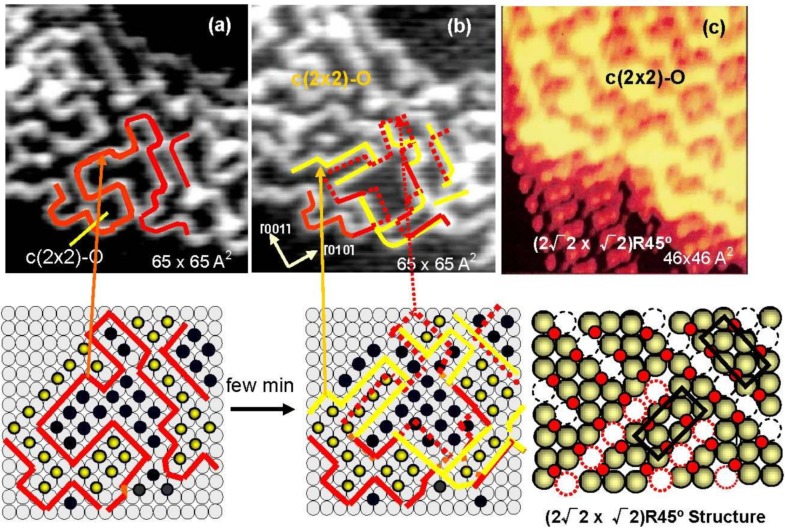
(**a**) STM image of a Cu(100) surface with 0.3 ML oxygen coverage, and (**b**) the STM image of the same area after few minutes at room temperature. The line drown in the image indicates the domain boundaries moved with time; (**c**) When a c(2 × 2)-O domain exceeds a critical size, the surface is relaxed by missing Cu atoms and a (2√2 × √2)R45° structure is established [34].

As shown in Figure 5(a) and Figure 5(b), domain boundaries move with time at room temperature, which proves migration of oxygen atoms from a nano c(2 × 2)-O domain to an adjacent c(2 × 2)-O nano-domain. It should be emphasized that O-atoms move but the c(2 × 2)-O nano-domain does not increase in size, that is, the surface covered with nano-c(2 × 2)-O domains is a stable state. From this dynamic behavior of the adsorbed O-atoms on the surface, the origin of the four-spot is the two out-phase nano-c(2 × 2)-O domains array regulated by local strain on the Cu(100) surface, which may increase non-linearly with the size of c(2 × 2)-O domain. Therefore, O-atoms migrate to keep the total energy lower, which is the origin of fluctuation of the domain boundaries with time. When oxygen coverage is increased, the c(2 × 2)-O domains should be in larger size, and a c(2 × 2)-O domain grows in a critical size lower the strain by mining Cu-atoms along the [001] or [010] directions, which is the mechanism of the formation of (2√2 × √2)R45° Cu(100)-O structure as shown in Figure 5(c). When the coverage reaches 0.5 ML, the Cu(100) surface is completely covered by the (2√2 × √2)R45° structure as shown in Figure 4(c) [33,34,35,36]. The (2√2 × √2)R45° Cu(100)-O surface is composed of the two domains according to the missing direction of Cu-atoms, and the two (2√2 × √2)R45° domains undergo rapid internal conversion by moving the Cu atoms from one site to an adjacent site within the Cu atoms missed ditches along [010] or [001] direction as illustrated by dotted circles in Figure 4.

**Figure 6:**
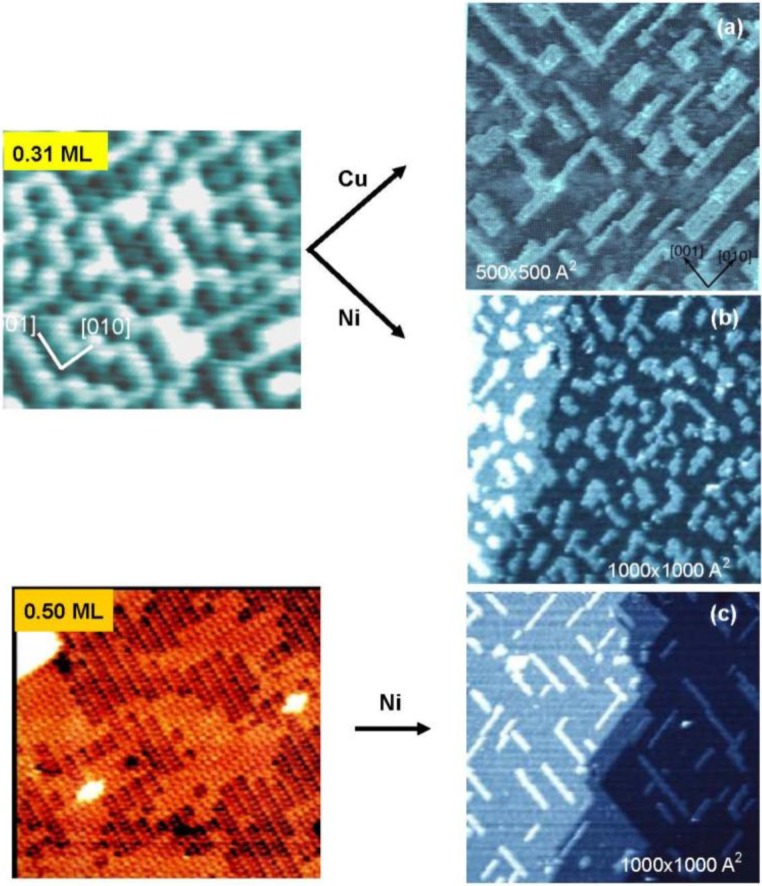
Surfactant growth of one atomic height (**a**) Cu layer and (**b**) Ni layer on a nano-c(2 × 2)-O Cu(100) surfaces [34,36]. The islands have rectangular shape of Cu-layer with Cu(100)-(2√2×√2)R45-O structure and non-oriented Ni-layer with Ni(100)-c(2 × 2)-O structure on a nano-c(2 × 2)-O Cu(100) surface; (**c**) Deposited Ni on on a Cu(100)-(2√2 × √2)R45-O surface grows in nano-width Ni-wires along the Cu atom missed ditches.

It is evident that adsorbed O-atoms are mobile on the four spots Cu(100)-O surface. Therefore, if Cu atoms deposit on a four spots Cu(100)-O surface, the adsorbed O-atoms play a surfactant role for the growth of the deposited Cu layer. As shown in Figure 6(a), rectangularly shaped islands are formed by a surfactant growth of Cu layer, that is, O-atoms make the growth of islands in a strain released (2√2 × √2) R45° structure. In contrast, when Ni-atoms were deposited on a four spots Cu(100)-O surface, growth of shapeless one atomic height Ni islands takes place as shown in Figure 6(b), where oxygen atoms on the four spots Cu(100) surface move onto the Ni layer surface by forming c(2 × 2)-O Ni(100). However, the Ni atoms deposited on a (2√2 × √2)R45° Cu(100)-O surface grow in nano Ni-wires along the Cu missed trenches, as shown in Figure 6 (c) [34,35,36].

## 3. Self-Assembly of Quasi-Compounds

Adsorption-induced restructuring has been widely studied on various single crystal surfaces by using various methods, but the restructuring process is still not clear. Important works providing insight into the restructuring process were presented by Ertl *et al.* [4,5] on an adsorption of oxygen on a Cu(110) surface, and by Besenbacher *et al.* [37] on a co-adsorption of H_2_ and O_2_ on a Ni(110) surface. They showed release of surface metal atoms and their rearrangement with adsorbed O or H atoms over the terrace in the presence of O_2_ or H_2_, and p(2 × 1)Cu(110)-O, p(3 × 1)Ni(110), and p(2 × 1)Ni(110)-O surfaces are formed by trapping Cu or Ni atoms and O-atoms at the terminal of (-Cu-O-) and (-Ni-O-) chains. To explain the growth of (-Cu-O-) or (-Ni-O-) chains, it is often explained as if migrating metal atom react with O-atoms at the terminal of (-Cu-O-) or (-Ni-O-) chains or on the terrace [7,8]. This explanation may be sound. The driving force for the release of metal atoms is a chemical reaction forming quasi-molecules such as (CuO)* and (NiO)*. Quasi-molecules are stabilized by forming quasi-compounds of (-Cu-O-) and (-Ni-O-) strings by weak internal bonding on the surface and the self-assembly of the quasi-compounds forms p(2 × 1)Cu(110)-O, p(3 × 1)Ni(110) and p(2 × 1)Ni(110)-O surfaces. This process is essentially different from the formation of c(2 × 2)-O structure by adsorbed O-atoms on Ni(100) and the formation of nano-c(2 × 2)-O domains on the Cu(100) surface and the formation of (2√2 × √2)R45° structure of Cu(100)-O surface by missing Cu atoms.

The idea of the formation of quasi-compounds or pseudo-molecules was first proposed by Tanaka [7,10] to explain the growth and array of (-Ni-O-) and (-Cu-O-) on Ni(110) and Cu(110) surfaces, and especially the (n × 1) array of (-Ag-O-) strings on Ag(110) surface. Now we have ample evidence for the existence of quasi-compounds, and we could design various new materials on the surface using this concept [38,39].

**Figure 7:**
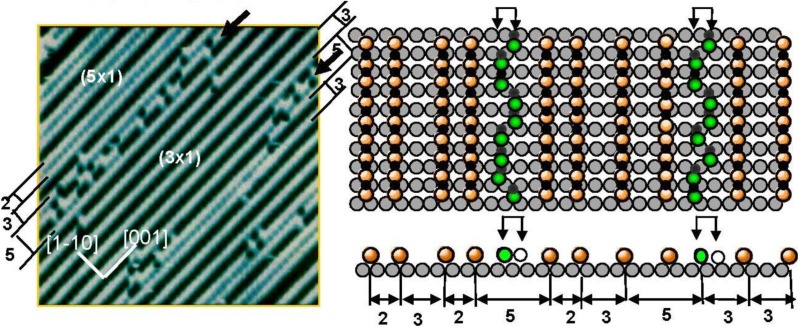
Self-assembly of (-Ag-O-) strings on a Ag(110) susrface make (n × 1) structure. A (-Ag-O-) chain along the phase bounday of the (3 × 1) domains undergoes fluctuation between the two energetically equivalent sites, which indicates weak internal bonding of (-Ag-O-) strings [41].

It was well known that the LEED pattern of Ag(110) surface exposed to O_2_ changed sequentially from p(7 × 1) to p(2 × 1) according to the oxygen coverage. This LEED pattern change was explained by the array of O-atoms on the Ag(110) surface [40]. However, the STM study proved it was not the ordered adsorption of O atoms but the growth in the (-Ag-O-) strings along the [001] direction. When the oxygen coverage was low, that is at a low concentration of (-Ag-O-) chains, the (-Ag-O-) strings are difficult to keep in a straight line, but as the (-Ag-O-) strings increase, they undergo self-assembly in the (n × 1) periodicity on the Ag(110) surface [9,10,11,13]. Fluctuation of (-Ag-O-) strings suggests their weak internal bonding. As the population of the (-Ag-O-) strings on the terrace increases, they form an ordered array in (nx1) structures and the value of “n” decreases sequentially from 7 to 2 as the population of (-Ag-O-) strings increases. In contrast to the (-Ag-O-) on Ag(110) surface, when a Cu(110) surface is exposed to O_2_, the (CuO)* quasi-molecules grow in a quasi-compound of (-Cu-O-) strings along the [001] direction, and the attractive interaction of the (-Cu-O-) strings undergoes formation of the (2 × 1) array on the Cu(110) surface. Accordingly, (-Cu-O-) strings make (2 × 1) islands on the Cu(110) surface, whereas (-Ag-O-) strings disperse in (nx1) structures as shown in Figure 7 [11,13,14]. The (5 × 1) domain of the (-Ag-O-) strings in this figure is composed of (2 × 1) + (3 × 1) phase, which has a higher density than that of the (4 × 1) and (3 × 1) phases. Dispersion of (-Ag-O-) strings in the [1-1 0] direction by making (n × 1) may be caused by the lattice strain induced by the arrangement of the (-Ag-O-) strings, which is similar to the strain induced by a large c(2 × 2)-O domain on Cu(100) surface. Another remarkable feature is the fluctuation of a (-Ag-O-) string along the antiphase domain boundary of the (3 × 1) phase. As shown in a model, two energetic degenerate sites appear in the phase boundaries, which are responsible for the fluctuation of a (-Ag-O-) string along the boundary. This phenomenon also suggests weak internal bonding of the (-Ag-O-) chain, but it can maintain a straight line in self-assembled ordered phase domains.

**Figure 8:**
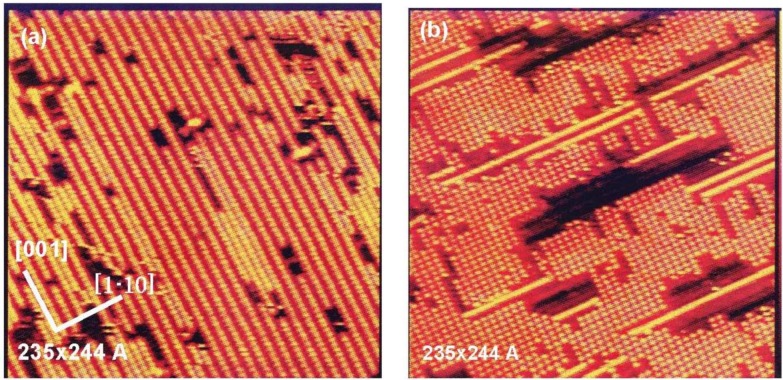
STM image indicating compression of (3 × 1) (-Ni-O-) phase by the adsorption of H_2_ on the Ni(110) surface at room temperature; (**a**) A p(3 × 1) Ni(110)-O phase (θ_o_ = 0.31 mL) on a Ni(110)-O; (**b**) The (3 × 1) (-Ni-O-) phase is compressed to (2 × 1) (-Ni-O-) phase by the growth of (-Ni-H-) strings along the [001] direction [12].

The idea of quasi-compounds suggests the possibility for new materials on specific surfaces [34,35,36,38,39]. In this respect, Besenbacher *et al.* [12] found an interesting phenomenon in the competitive growth of (-Ni-O-) and (-Ni-H-) on a Ni(110) surface, on which (3 × 1) (-Ni-O-) strings was compressed to (2 × 1) array by the adsorption of H_2_ at room temperature, as shown in Figure 8. This phenomenon is difficult to explain by the traditional idea of the adsorption of O and H (adsorption of O is far stronger than that of H), but is well explained by the competitive adsorption of quasi-molecules of (NiH)* and (Ni-O)* and the growth (Ni-H)* in the (-Ni-H-) strings, which is a driving force to compress the (3 × 1) (-Ni-O-) strings to the (2 × 1) arrangement.

**Figure 9:**
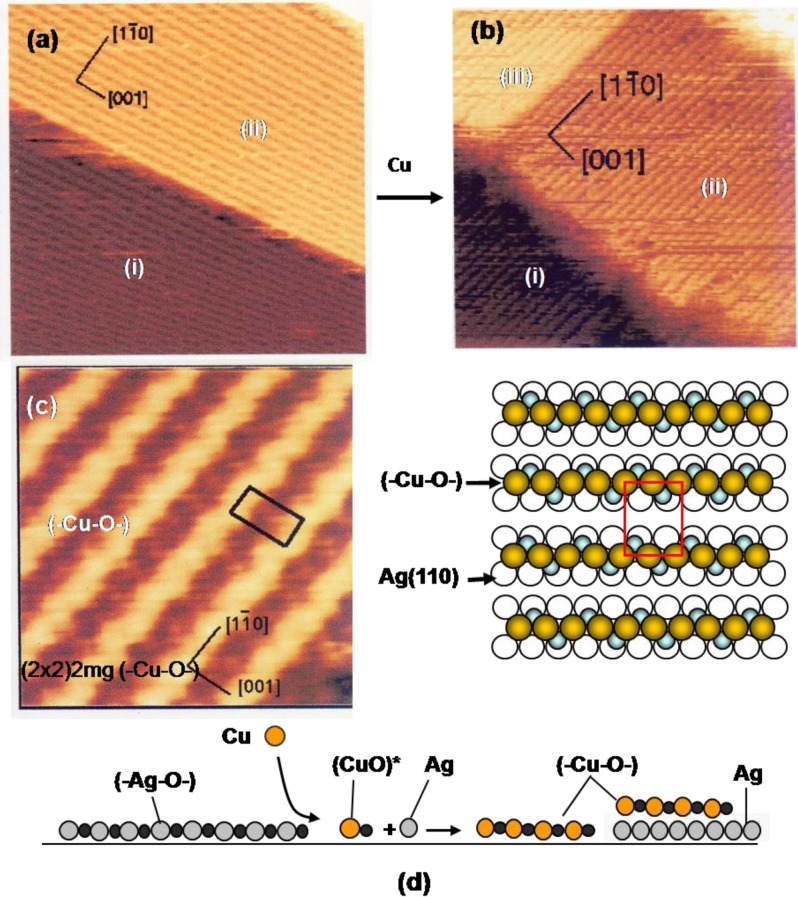
(**a**) A Ag(110) surface covered with the (2 × 1) (-Ag-O-) strings; (**b**) Depositing Cu atoms on a (2 × 1) (-Ag-O-) Ag(110) surface, (-Cu-O-) strings are formed by the reaction with (-Ag-O-) strings, Cu + (-Ag-O-) → Ag + (-Cu-O-), and the (-Cu-O-) strings grow by making a (1 × 2) structure. Released Ag atoms make a new terrace-(iii), which is also covered with (-Cu-O-) strings; (**c**) A high resolution STM image proves zig-zag structure of the (-Cu-O-) strings on the Ag(100) surface, which is expressed by the (2 × 2)2 mg [43,45]; (**d**) Illustration of the reaction of the (-Ag-O-) strings with Cu atoms.

It is noteworthy that the oxygen adsorbed on the Cu(100) surface acts as a surfactant in the growth of mono-atomic layers of Cu or Ni, as shown in Figure 6, but the growth of (-Cu-O-) chain, on Cu(110), (-Ni-O-) chain, on Ni(110), and (-Ag-O-) chain, on the Ag(110) surface are evidently different from the surfactant growth of metal layers on the nano-c(2 × 2)-O Cu(100) surface. The idea of quasi-compounds is quite valuable to rationalize various phenomena taking place on the surface. Growth of (-Cu-O-) strings on a Ag(110) surface is a typical example. When Cu atoms deposit on a Ag(110) fully covered by (-Ag-O-) strings in the (2 × 1), the LEED pattern changes from p(2 × 1) to (2 × 2)-p2 mg. The STM image proves a dramatic construction of the surface by deposited Cu atoms as shown in Figure 9(a) to Figure 9(d). That is, the reaction of Cu atoms with (-Ag-O-) strings creates (-Cu-O-) strings along the [1-1 0] direction on the terrace (i) and (ii), and on a newly formed terrace (iii) by released Ag atoms [42,43,44]. This result suggests the formation of (CuO)* quasi-molecules by the reaction of (-Ag-O-) strings with Cu atoms, and (CuO)* growth in (-Cu-O-) strings. It is noteworthy that the edges of the terrace (i), (ii) and (iii) run straight along the [001] and [1-1 0] directions, which suggests the self-assembly of the (-Cu-O-) strings provided by the reaction of the (-Ag-O-) with Cu atoms.

While the (-Cu-O-) chain runs straight on Cu(110) surface along the [001] direction, the (-Cu-O-) strings grown on the Ag(110) surface have a zigzag structure as shown by a high resolution STM image. The LEED pattern of (2 × 2)-p2 mg is well explained by the in-phase zigzag structure when they are arranged in (1 × 2) as shown in Figure 9(c). However, the zigzag structure is difficult to keep in-phase when the (-Cu-O-) strings are arranged in the (1 × 3) structure. This is an order-disorder change caused by a weak mutual interaction [43,45].

**Figure 10:**
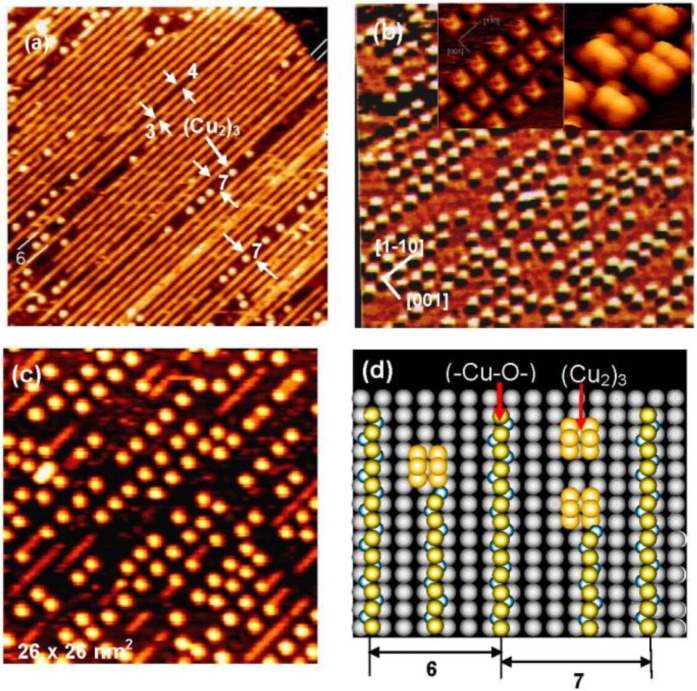
(**a**) The (1 × 3) (-Cu-O-) strings arrayed on a Ag(110) surface undergo decomposition along the phase boundary having seven lattice spacing at 570 °K, and (Cu_2_)_3_ dots are formed [43]; (**b**) An ordered array of (Cu_2_)_3_ dots formed by complete decomposition of (-Cu-O-) strings on a Ag(110) surface. A high resolution inset STM image suggests a structure of (Cu_2_)_3_, and a lone Cu_2_ is also seen; (**c**) Reverse change of (Cu_2_)_3_ dots to (-Cu-O-) strings occurs by exposing to O_2_ at room temperature; (**d**) A model suggesting preferential decomposition of (-Cu-O-) strings to (Cu_2_)_3_ along the phase boundary.

We can expect new properties of the (-Cu-O-) strings formed on the Ag(110) surface. As a matter of fact, the (-Cu-O-) strings formed on Ag(110) readily decompose at low temperatures such as ca. 500 °K, and uniform-sized square shape dots are formed, as shown in Figure 10(a), which is a remarkable contrast to the stability of the (-Cu-O-) chains on Cu(110). An interesting feature observed on the decomposition of the (1 × 3) (-Cu-O-) strings on the Ag(110) surface is that the decomposition proceeds preferentially along a domain boundary in the (1 × 3) phase, which has a four lattice spacing. The decomposition of a (-Cu-O-) string produces uniform-sized square shape dots in a 7 lattice spacing (3 + 4) along the [1-1 0] direction. The decomposition of (-Cu-O-) strings was complete in about 5 min at 570 °K, which gave an ordered arrangement of uniform-sized square shape Cu-dots on the Ag(110) surface, as shown in Figure 10(b). Inset images in Figure 10(b) show the fine structure of the square shaped dots seen by the STM, which suggests the (Cu_2_)_3_ structure ( a fragmentary Cu_2_ is also seen).

When the (Cu_2_)_3_ dots on a Ag(110) surface are exposed to O_2_, (-Cu-O-) strings sprout from a corner of the square (Cu_2_)_3_ dots as shown in Figure 10(c). The decomposition of the (-Cu-O-) strings to (Cu_2_)_3_ dots and the regeneration of (-Cu-O-) strings from the (Cu_2_)_3_ dots are schematically shown in Figure 10(d), which explains well the preferential decomposition of a (-Cu-O-) string in the (Cu_2_)_3_ dots along the phase boundary with a seven lattice space. That is, the (Cu_2_)_3_ dots stay in a six lattice spacing when they are formed by the decomposition of a (-Cu-O-) string inside the (1 × 3) phase. The (Cu_2_)_3_ dot formed in the seven lattice space takes an equal distance from the (-Cu-O-) strings of each side, but the (Cu_2_)_3_ dot formed in the six lattice space takes different distances from the (-Cu-O-) strings of each side. This result is an interesting piece of evidence showing that different reaction spaces give different reactivity, that is, the reaction spacing is responsible for the preferential decomposition of (-Cu-O-) strings along the phase boundary of the (1 × 3) domains. It might be a similar effect observed in Figure 2, where the mutual distance of the -CH_3_ groups in the domain boundaries influences the array of molecules.

**Figure 11:**
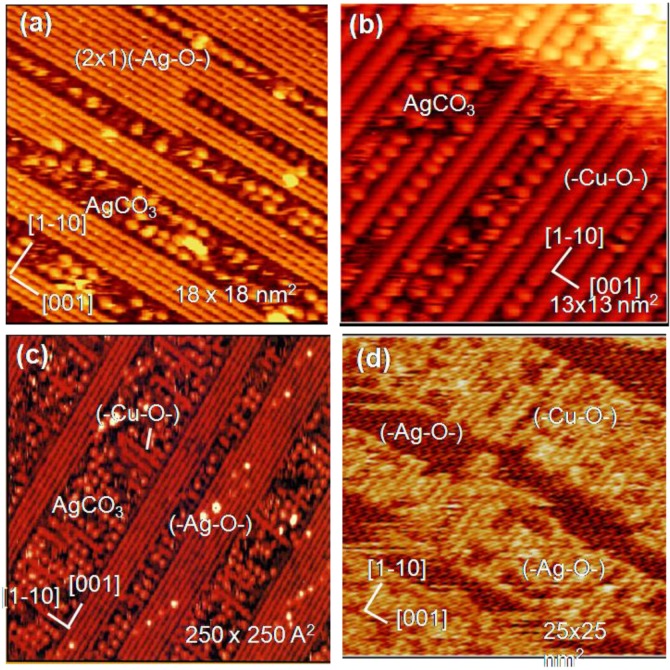
Selective reaction of (-Ag-O-) strings on the Ag(110) surface; (**a**) When a (3 × 1) (-Ag-O-) Ag(110) is exposed to CO_2_, the (3 × 1)(-Ag-O-) phase is compressed to a (2 × 1) phase by forming Ag-CO_3_ dots; (**b**) Cu atoms deposited on a composite Ag(110) surface of (-Ag-O-) strings and Ag-CO_3_ dots undergo selective reaction with the (-Ag-O-) strings to form (-Cu-O-); (**c**) By scanning a composite Ag(110) surface by a Cu/W-tip, the Cu atoms transferred from the W-tip react selectively with AgCO_3_ dots to form (-Cu-O-) strings. The first sweep of the surface by a Cu/W-tip shows in (c) and after the ten times sweep in ca. 2 min is shown in (**d**) [38,46,9].

Another interesting example is the reaction of (-Ag-O-) strings with CO_2_. When (3 × 1) (-Ag-O-) Ag(110) surface is exposed to CO_2_, (-Ag-O-) reacts with CO_2_ and forms \ Ag-CO_3_ dots, and the dots compresse the (3 × 1) (-Ag-O-) phase to (2 × 1)(-Ag-O-) phase, as shown in Figure 11(a), that is, a reaction of (-Ag-O-) + CO_2_ → Ag-CO_3_ compresses the arrangement of (-Ag-O-) [46]. Interestingly, when Cu atoms deposit on the composite surface of (2 × 1) (-Ag-O-) strings and AgCO_3_ dots, Cu atoms react selectively with the remaining (-Ag-O-) strings and (-Cu-O-) strings grow along the [1-1 0] direction. As (-Cu-O-) strings grow in perpendicular to the (-Ag-O-) strings, the (-Cu-O-) strings make the AgCO_3_ dots redistribute on the surface as shown in Figure 11(b). When this newly formed composite surface of (-Cu-O-) strings and Ag-CO_3_ dots was scanned by a W-tip contaminated with Cu-atoms, Ag-CO_3_ dots reacted with the Cu atoms from the W-tip but (-Cu-O-) strings do not react. The observed curious selective reaction of Cu atom with quasi-compounds is described as follows:

1) (3 × 1)(-Ag-O-) + CO_2_(g) → (2 × 1)(-Ag-O-) + AgCO_3_
2) (2 × 1)(-Ag-O-) + Cu → (-Cu-O-) + Ag
3) AgCO_3_ + Cu/W-tip → (-Cu-O-) + Ag + CO_2_

where **underline** indicates the compounds formed on the Ag(110) surface.

It is clear that deposited Cu atoms react with (-Ag-O-) but are inactive to Ag-CO_3_ dots. In contrast, Cu atoms provided from the W-tip react selectively with Ag-CO_3_ dots as shown in Figure 11(c) and Figure 11(d) [9]. Different selectivity between the deposited Cu atoms and the Cu atoms provided from the W-tip might be due to the local potential given by the STM tip. Different reactivity of Cu atoms is illustrated in the following reaction scheme.

**Scheme 1:**
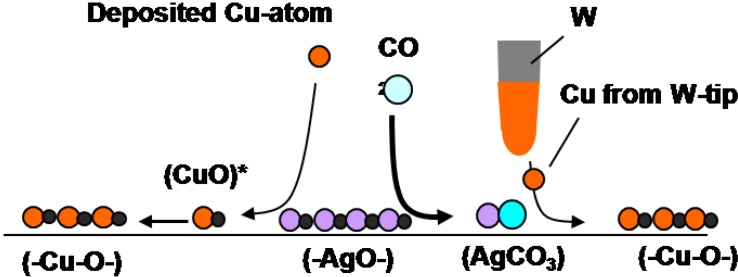
Reaction scheme of quasi-compounds**:** Reaction Cu atoms with (-Ag-O-) and Ag-CO_3_ on Ag(110) is quite different by the deposition way of Cu atoms. Cu atoms deposited by vaporization react selectively with (-Ag-O-) strings but Cu atoms transferred from a Cu-W-tip reacts selectively with AgCO_3_ but not with (-Ag-O-).

## 4. Formation of Nano-Composite Surfaces

Patterning by the self-assembly of adsorbed atoms, molecules, and quasi-compounds has been discussed above. As discussed in this section, stable compounds formed on the surface give another type of patterning.

**Figure 12:**
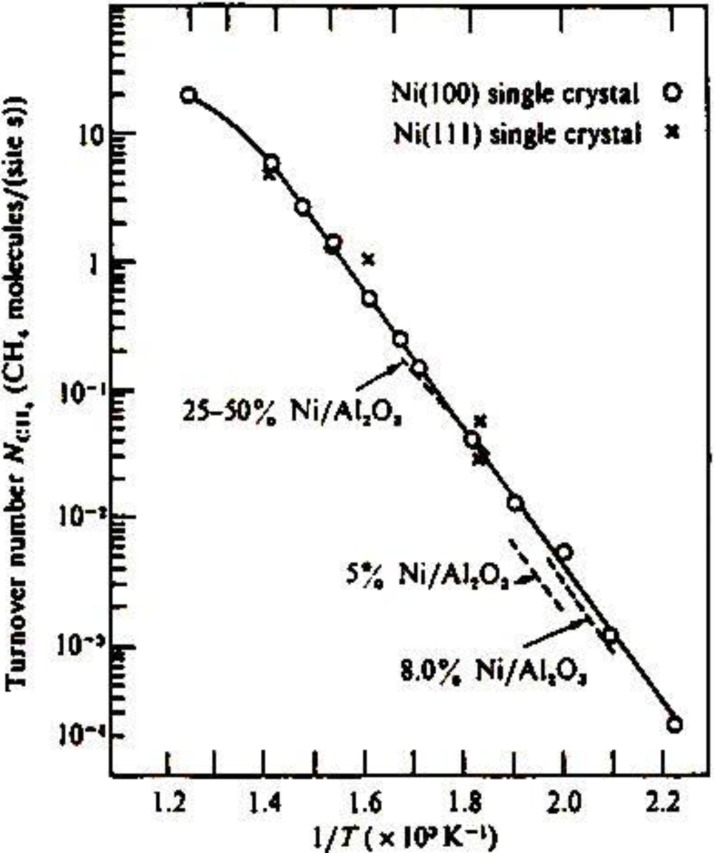
Structure independent turn-over frequency of various forms of Ni-catalyst for the methanation reaction, 3 H_2_ → CH_4_ + H_2_O, [47].

The methanation reaction, CO + 3 H_2_ → CH_4_ + H_2_O, is catalyzed by Ni catalysts. The catalytic activity of Ni is known to be structure insensitive, that is, the turnover frequency of the formation of CH_4_ per Ni-site is not influenced by the morphology of Ni, either single crystal or supported particles, and the concentration of Ni on support as shown in Figure 12(a) [47]. This characteristic activity has been explained by the reaction mechanism, that is, an intermediate carbon (C) is formed by a rapid disproportionation reaction of CO, 2 CO→CO_2_ + C, and its hydrogenation to methane, C → CH_x_ → CH_4_, is the rate determining step. What carbon intermediates are formed on the Ni catalyst?

**Figure 13:**
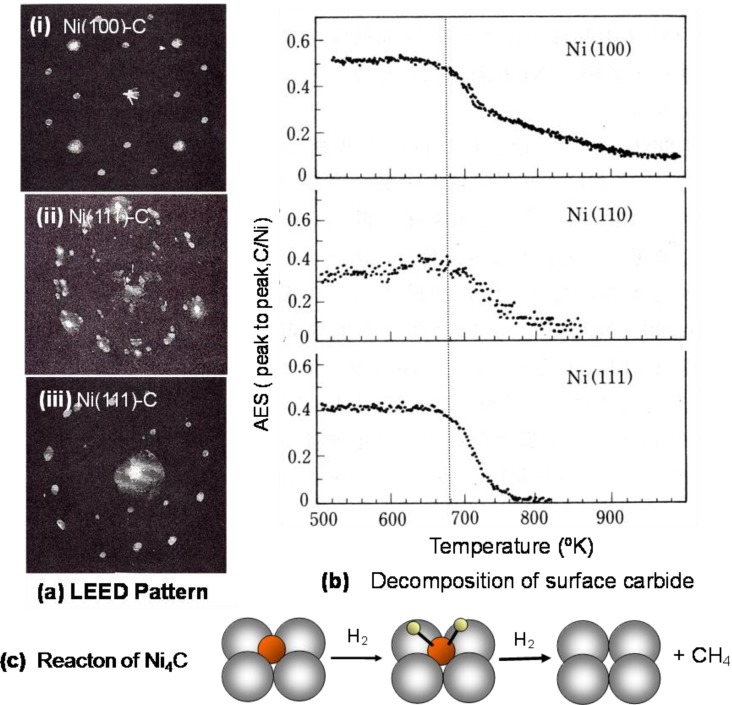
(**a**) LEED pattern of carbon segregated on Ni(111), Ni(100) and Ni(110) surfaces; (**b**) Decrease of segregated carbon on Ni(111), Ni(100) and Ni(110) surfaces by heating in vacuum; (**c**) Structure insensitive methanation is explained by the hydrogenation of a common intermediate of Ni-carbide. Steady state Ni-carbide given by a dynamic balance of the formation of Ni-carbide and its hydrogenation is similar on various Ni-catalysts [49,50].

When a single crystal surface of Ni(111), Ni(100), or Ni(110) is heated in an ultra high vacuum, the surface is covered with segregated impurity carbon, and a characteristic LEED pattern described as (2 × 2)p4g-Ni(100)-C, c(4 × 5)-Ni(110)-C, and (√39 × √39)-Ni(111)-C such as shown in Figure 13 appears. As discussed below, these LEED patterns reflect the array of carbide molecules on the crystal surfaces. The LEED pattern of Ni(111)-C surface is complex because of the coexistence of three domains. The diffraction pattern of a single domain area of the array of carbide on a Ni(111) surface is shown in Figure 13 as a LEED pattern (iii) [48].

When a surface of Ni(100)-C, Ni(110)-C, or Ni(111)-C is heated under vacuum, carbon atoms disappears at a temperature almost equal to 680–690 °K according to very similar profiles as shown in Figure 13(b). Why do the carbon atoms segregated on the surface decrease from the surface by heating? Stabilization of Ni-carbide on Ni surface is a driving force for the segregation of carbon atoms because the formation of Ni-carbide is unstable in the Ni bulk due to the large lattice strain. The Ni-carbide formed on the Ni surfaces has an equal decomposition, so that the Ni-carbide formed on a single crystal surface undergoes decomposition at almost the same temperature on Ni(100), Ni(110), and Ni(111) surfaces. The carbon atom formed by the decomposition of a Ni-carbide molecule dissolves into the bulk of Ni crystal instead undergoing desorption. According to this mechanism, the hydrogenation of nickel carbide (Ni_4_C) provided by the disproportionation of CO is responsible for the structure independent catalysis [49,50].

**Figure 14:**
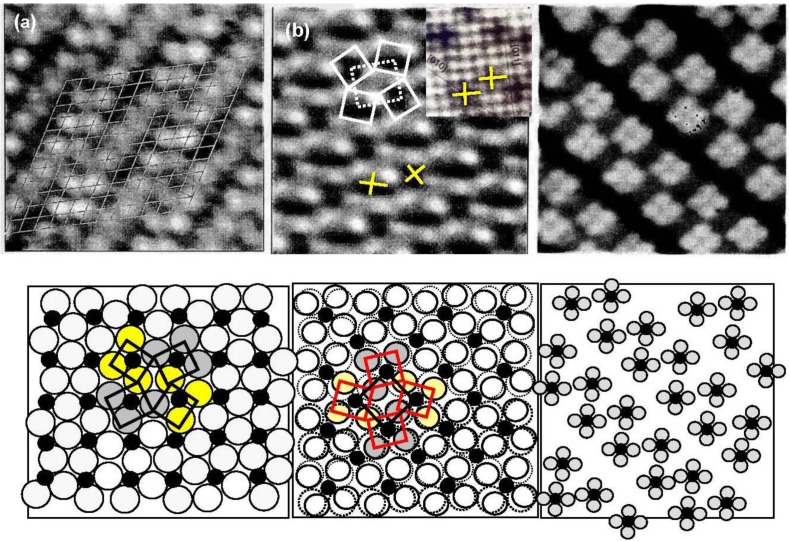
STM images of Ni-carbide formed on Ni(111), Ni(100) and Ni(110) surfaces [51,52] ; (**a**) (√39 × √39)-C/Ni(111); (**b**) (2 × 2)p4g-C/Ni(100). Inset image shows random segregation of Ni_4_C molecules in an initial; (**c**) c(4 × 5)-C/Ni(110) surface.

Besenbacher and his coworkers [51] showed the growth of Ni-carbide by the STM on Ni(100), Ni(110) and Ni(111). The inset STM image of Figure 14(a) shows that the Ni_4_C carbide molecules (dark crosses) are randomly formed on the Ni(100) surface, in which a dark spot at the four-fold hollow site on the Ni(100) surface does not represent adsorbed C atom but rather the formation of an inlaid Ni_4_C molecule on the Ni(100) surface. As the inlaid Ni_4_C molecules of the Ni(100) surface increase, the carbide molecules (Ni_4_C) undergo rotation alternately in clockwise and counterclockwise direction on the Ni(100) surface to reduce the lattice distortion, as shown in Figure 14(b). The complex structures of the Ni(111)-(√39 × √39)-C surface and the Ni(110)-c(4 × 5)-C surface are explainable by the array of the Ni_4_C carbide molecules. It should be pointed out that the amount of Ni_4_C on Ni catalyst during catalysis is given by a dynamic balance of the formation of Ni_4_C and its hydrogenation to CH_4_.

**Figure 15:**
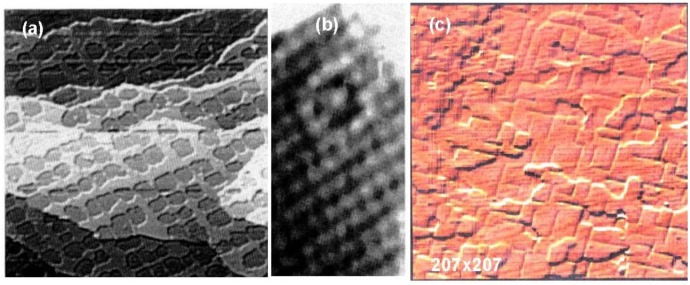
(**a**) A Cu(100) surface covered with c(2 × 2)-N patches; (**b**) a high resolution image of a c(2 × 2)-N patch of ca. 5 × 5 nm^2^ [57]; (**c**) A Cu(100) surface fully covered with c(2 × 2)-N layer [82].

Analogous phenomenon of the formation of surface compounds is observed when the Cu surface is bombarded with nitrogen ions. The N_2_ molecule cannot dissociate on the Cu surface, but Cu-nitride is formed by bombarding the Cu surface with nitrogen ions. Uptake of N atoms is thus often described as adsorbed N-atoms on the surface. As it is discussed below, uptake of N atoms is not due to the adsorption of N-atoms but rather the formation of Cu-nitride on the surface. When a Cu(111) surface is bombarded with N-ions at 300 °K, Cu-nitride is randomly formed over the surface. By annealing this surface at 500 °K, the Cu(111) surface is covered with three domains of Cu(100)-c(2 × 2)-N-like layers [53,54]. Similarly, if a Cu(110) surface bombarded with nitrogen ion is annealed at 650 °K, the Cu(110)-(2 × 3)-N surface is established. The structure of Cu(110)-(2 × 3)-N was precisely studied using fully dynamical LEED by Bradshaw and his coworkers [55], and they deduced that a pseudo Cu(100)-c(2 × 2)-N layer was formed on a corrugated Cu(100)-like interlayer of the Cu(110) surface. Therefore, the structure of the topmost layer of the Cu(110)-(2 × 3)-N surface is very similar to the Cu(100)-c(2 × 2)-N surface, which is similar to the Cu_3_N(100) plane [55,56]. These results suggest that the Cu(100), Cu(110), and Cu(111) surfaces are covered with an epitaxially grown Cu_3_N(100) plane. The growth of the Cu_3_N(100) plane on the Cu(100) surface was shown by Leibsle *et al.* [57] using STM. When a Cu(100) surface bombarded with nitrogen ions is annealed at 600 °K, uniform size square patches (5.2 ± 0.4 nm^2^) taking a Cu(100)-c(2 × 2)-N structure are formed on the Cu(100) surface, as shown in Figure 15(a). They pointed out that the Cu-Cu distance of the Cu_3_N (100) plane is about 5% longer than that of the Cu(100) surface. Based on this mismatch, they speculated that 14 × 4 = 56 Cu atoms might be ejected around an inlaid c(2 × 2)-N patch in the Cu(100) surface. On the other hand, the LEED pattern suggested a smaller than 0.05% lattice mismatch of the c(2 × 2)-N layer on the Cu(100) surface, and Komori and his coworkers [58] showed the accumulation of strain in the vicinity of the c(2 × 2)-N patches by atomically resolved STM. From this result, they proposed an inhomogeneous lattice distortion caused by adsorption of N in c(2 × 2)-N on the Cu(100) surface. If the square patch formed on Cu(100)-N is not the adsorption domain of c(2 × 2)-N but a stable Cu_3_N(100) inlaid in the Cu(100) surface, the lattice distortion of the Cu(100) surface will be also largest at the vicinity of the inlaid 5 × 5 nm^2^ patches of Cu_3_N(100) plane. The lattice distortion may occur similarly on either the domain of adsorption or the compound domain. As shown in Figure 5, the size of c(2 × 2)-O domains is regulated by the strain, where O-atoms migrate from one c(2 × 2)-O domain to a neighbor C(2 × 2)-O domain to maintain the size of c(2 × 2)-O domains. In the case of compounds, no such migration as observed in the adsorption may occur but the size of the domain is decided in the preparation. Therefore, the Cu(100)-(2 × 2)-N surface is made by inlaying stable Cu_3_N(100) plane in the Cu(100) surface, which may be different from the adsorption of N atoms at the four-fold hollow sites on the Cu(100) surface. We conclude that the nano-size Cu_3_N(100) plane is inlaid or overlaid on the Cu(100), Cu(111) and Cu(110) surfaces, which is similar to the formation of the Ni(100), Ni(111) and Ni(110) surfaces covered with inlaid Ni_4_C molecules shown in Figure 14.

**Figure 16:**
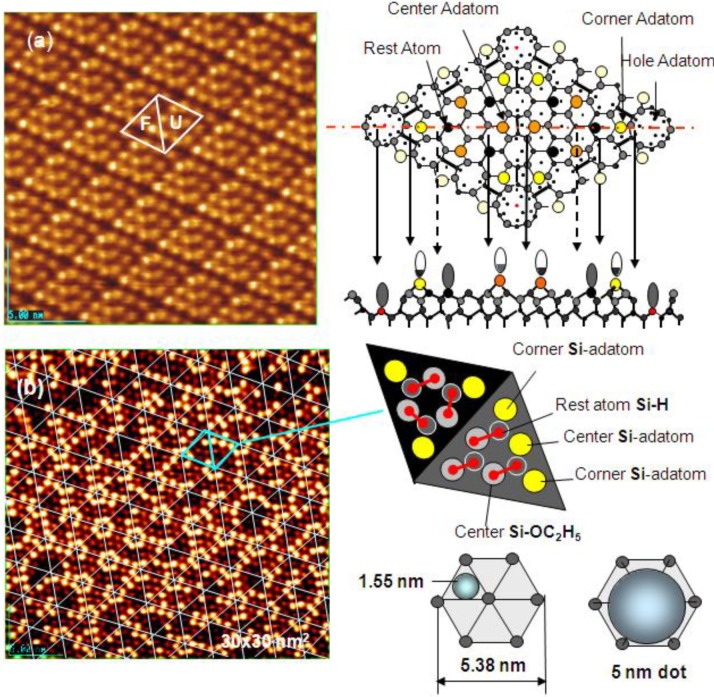
(**a**) A STM image of a clean Si(111)-7 × 7 surface. F and U are the faulted and unfaulted half-unit cells. A model of Si(111)-7 × 7 surface and the electron density of the dangling bonds; (**b**) A STM image of a Si(111)-7 × 7 surface saturated with C_2_H_5_OH (θ = 0.49). Dissociation of C_2_H_5_OH on (Si-adatom-Sirest atom) pair site is shown with bar [59]. Estimation of the growth of dot in a half unit cell (1.5 nm) and in a hexagonal cell (5 nm) is illustrated.

As discussed in the next section, the composite surface made by stable compounds constitutes a new functional surface for the preparation of nano-materials. Here we show another type of composite surface prepared on a Si(111)-7 × 7 surface by a chemical modification. It is known that the cleaved Si(111) surface is not a stable surface so that it reconstructs to a stable Si(111)-7 × 7 surface by annealing as shown in Figure 16(a). A unit cell of the Si(111)-7 × 7 surface is composed of triangular faulted and unfaulted half unit cells. A half unit cell has nine unsaturated Si-atoms, six Si-adatoms and three Si-rest atoms, which have a dangling bond, as described schematically in Figure 16.

When the Si(111)-7 × 7 surface is exposed to CH_3_OH or C_2_H_5_OH (ROH), the ROH molecule dissociates on the pair site of (Si-adatom/Si-rest atom) as described by an equation [59]:

*Si-adatom/Si-rest atom) + ROH → (RO-Si-adatom/H-Si-restatom)*

When the Si(111)-7 × 7 surface is saturated by the adsorption of C_2_H_5_OH (hereafter denoted Si(111)-7 × 7-C_2_H_5_OH), a half unit cell is composed of three C_2_H_5_O-Si-adatoms, three H-Si-rest atoms, and three intact Si-adatoms. Figure 16(b) shows the STM image of a Si(111)-7 × 7-C_2_H_5_OH, the three bright spots in each half unit cell are the intact Si-adatoms.

**Figure 17:**
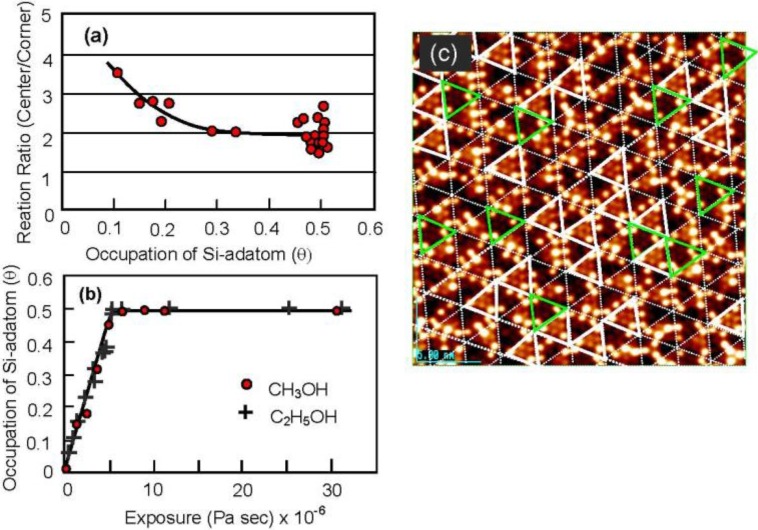
(**a**) Dissociation probability of CH_3_OH on (corner Si-adatom/rest Si-atom) is four time larger than that on (center Si-adatom/rest Si-atom) pair site; (**b**) Dissociation probability of CH_3_OH and C_2_H_5_OH on the Si(111)-7 × 7 surface is coverage independent; (**c**) Solid triangles are the half unit cells having three corner Si-adatoms [60,61].

By counting the dark spots, we can deduce the dissociation probability of C_2_H_5_OH on the Si-adatoms. As shown in Figure 17(a) and Figure 17(c), the dissociation on center-adatom/rest-atom pair sites is four times larger than that on the Si corner-adatom/rest-atom pair sites. This difference is explained well by the conformation of the pair sites, that is, a center Si-adatom is adjacent to two Si-rest atoms but a corner Si-adatom is adjacent to one Si-rest atom [60]. The total sticking probability of CH_3_OH and C_2_H_5_OH on the Si(111)-7 × 7 surface, however, is coverage independent as shown Figure 17(b), which strongly suggests that the dissociation of alcohol molecules takes place via an irreversible precursor state [60,61].

It should be pointed out that the Si(111)-7 × 7 surfaces saturated with C_2_H_5_OH are inactive for the dissociation of alcohol molecules but have three intact Si-adatoms in every half unit cell. These intact Si-adatoms will act as active sites and/or the nucleation sites for the growth of materials as shown in the next section.

## 5. Controlled Growth of Nano-Materials on Composite Surfaces

As shown in Figure 4(c), when the oxygen coverage on a Cu(100) surface exceeds a certain value, Cu-atoms are removed from the surface along the [001] and the [010] direction, and the missing row structure is completed over the surface at a coverage of 0.5 ML. When Ni is vaporized on a (2√2 × √2)R45° Cu(100)-O surface made by removal of Cu-atoms, nano-width Ni-lines grow along the missing Cu-atom ditches as shown in Figure 6(c). In this case, the Ni-atom trapped in a ditch may act as a nucleation site for the growth of Ni nano-wires. This phenomenon is a kinetic controlled growth of nano-materials on a composite surface.

**Figure 18:**
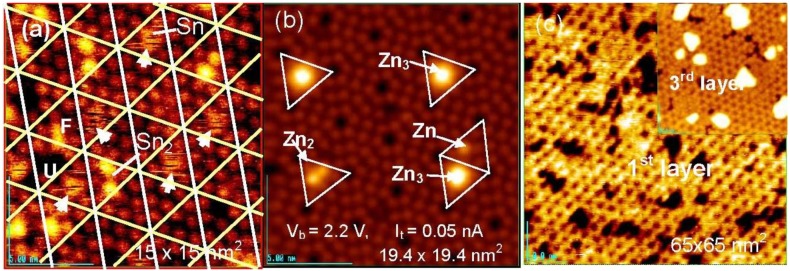
Sn and Zn atoms deposited on a clean Si(111)-7 × 7 surface at room temperature; (**a**) Fuzzy image indicates a rapidly migrating Sn-atom in a half unit cell. When a 2nd Sn atom comes in a half unit cell, migration of Sn atoms stops [62]; (**b**) Initial stage of deposited Zn, Zn_2_ and Zn_3_ localized on the center Si-adatoms; (**c**) Honeycomb over-layer composed of Zn_3_-cluster formed on a Si(111)-7 × 7 surface. An inset image (45 × 45 nm^2^) shows the supra-honeycomb structure of the 3rd layer [64].

From this viewpoint, the adsorption and the growth of metal dots on a clean Si(111)-7 × 7 surface and on a Si(111)-7 × 7-C_2_H_5_OH surface were studied. As shown in Figure 16(a), six bright Si-adatoms (three corner and center Si-adatoms) are seen in every half unit cell on the clean Si(111)-7 × 7 surface by the STM, but three rest atoms are invisible. When metal atoms deposit on a clean Si(111)-7 × 7 surface, two different kinds of selective adsorption occurs; one is the selection of faulted half (F) or unfaulted (U) half unit cells, and the other is the selection of center Si-adatoms or corner Si-adatoms in a half unit cell. It is known that most metals (Tl, Li, K, Na, Pb, Y, Cu, Ag, and Au) prefer the faulted half unit cell, but Sn [62,63], In [63], and Zn [64] adsorb on both the faulted and unfaulted halves. Metals forming no silicide are stabilized by adsorbing on the Si-adatoms with a dangling bond. As shown in Figure 18 (b), Zn atoms are stabilized on the center Si-adatoms in a Zn, Zn_2_, and Zn_3_ form.

In the case of the adsorption of Sn atoms, however, a single Sn-atom trapped in a half unit cell gives a fuzzy image, as shown in Figure 18(a), which suggests rapid movement of the Sn atom within a half unit cell. However, when a second Sn atom comes into the same half unit cell, the migration of the Sn atoms stops and a clear image of the two Sn atoms is seen, as shown in Figure 18(a) [62]. This result indicates that the hopping migration of Sn atoms is frozen from occupying an adjacent Si-adatom by the adsorption of a second Sn atom. Similar rapid migration was reported for Pb [65], Ag [66,67], and Y [68]. Tsong *et al.* [66] proposed the contribution of the Si-rest atoms in the hopping migration of Ag atom. If Si-rest atoms contribute to the migration of a metal atom on a Si(111)-7 × 7 surface, the dynamic behavior of metal atoms on the Si(111)-7 × 7-C_2_H_5_OH surface is quite interesting because all the whole Si-rest atoms are changed to H-Si-rest atoms. At the same time, metal atoms will be adsorbed on the intact Si-adatoms on the Si(111)-7 × 7-C_2_H_5_OH surface, and they will work as nucleation sites for the growth of dots. If the dots will grow on this surface as expected, when metal atoms are supplied to the dots, which is the direct collision or surface diffusion?

**Figure 19:**
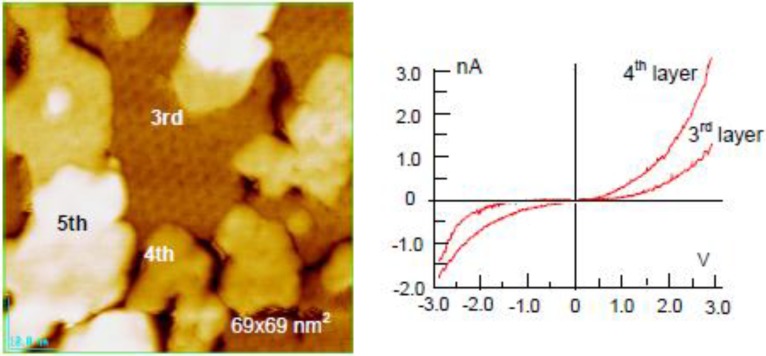
The 3rd supra-honeycomb layer has a band gap but the 4th and 5th Zn layers are metallic on a Si(1111)-7 × 7 surface [64].

Clustering of metals on clean Si(111)-7 × 7 surfaces has been widely studied, including Sn [62,63], In [63], Zn [64], Pb [63,65,69], Ag [70,71], Tl [72], Al [73], and Ga [74,75]. In a case of Zn atoms, these adsorb on the center Si-adatoms Si(111)-7 × 7 surface in a form of Zn, Zn_2_, and Zn_3_ at very low coverage, as shown in Figure 18(b). When the coverage of Zn is increased, the Si(111)-7 × 7 surface is covered by a Zn_3_-cluster in every half unit cell, which forms a honeycomb structure composed of Zn_3_ clusters as shown in Figure 18(c). More deposition of Zn atoms makes the 2nd and 3rd layers grow by maintaining the honeycomb structure, in which a Zn_3_ cluster stacks just on the Zn_3_ cluster of the layer beneath by rotating 60 degrees. A honeycomb hole at a center of six Zn_3_ clusters on the topmost layer become shallower and shallower as the layers increase, and the honeycomb structure cannot be maintained in the 4th and 5th layers, as shown in Figure 19. The electronic properties also change between the 3rd layer and the 4th layer, that is, from a semi-conductive layer to a metallic layer as shown by tunneling spectroscopy in Figure 19 [64].

**Figure 20:**
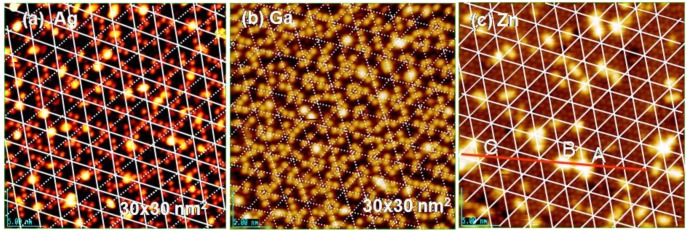
Single atom of Ag, Ga, and Zn adsorbed on a Si(111)-7 × 7-C_2_H_5_OH surface; (**a**) Ag-atoms are stabilized on either corner or center Si-adatoms; (**b**) Ga-atoms are stabilized on corner Si-adatoms, and (**c**) Zn-atoms prefers to form pairs on two neighboring Si-adatoms [71].

The dynamic behavior of metal atoms on the Si(111)-7 × 7-C_2_H_5_OH surface is entirely different from that on the clean Si(111)-7 × 7 surface. For example, a Ag atom on a clean Si(111)-7 × 7 surface may migrate by hopping on the Si-adatoms via Si rest-atoms [76], and Zhang *et al.* [67] deduced that Ag atoms adsorbed on Si(111)-7 × 7 surfaces prefer the corner Si-adatms in faulted half cells but on the center Si-adatoms in unfaulted half cells at 298 °K , which is an equilibrium distribution. If the hopping migration of Ag atoms is prohibited, Ag atoms may adsorb randomly on Si-adatoms, and this it is an equilibrium distribution. In this respect, adsorption of Ag atoms on the Si(111)-7 × 7-C_2_H_5_OH surface is quite interesting, because half of the Si-adatoms (three) are intact although three other Si-adatoms and all the Si-rest atoms (three in a half unit cell) are occupied by forming C_2_H_5_O-Si, but H-Si-rest on the Si(111)-7 × 7-C_2_H_5_OH surface as shown in Figure 16(b). Therefore, Ag atoms adsorbed on the Si(111)-7 × 7-C_2_H_5_OH surface cannot migrate by hopping via Si-rest atoms. As a result, adsorption of Ag atoms will occur randomly on the intact corner and center Si-adatoms. In fact, single Ag atoms are randomly adsorbed on the intact Si-adaoms on Si(111)-7 × 7-C_2_H_5_OH surface as shown in Figure 20(a) [71]. Adsorption of Ga and Zn atoms occurs in a similar manner on the Si(111)-7 × 7-C_2_H_5_OH surface, as shown in Figure 20(b) and Figure 20(c), on which bright Ga and Zn atoms coincide with the intact Si-adatoms on the Si(111)-7 × 7-C_2_H_5_OH surface. Therefore, we could conclude that deposited metal atoms are frozen in a single atom on an intact Si-adatom of the Si(111)-7 × 7-C_2_H_5_OH surface

Intact Si-adatom remaining on the Si(111)-7 × 7-C_2_H_5_OH surface is composed of a ratio of (corner-Si-adatom/center-Si-adatom) ≈ 2, because dissociation of C_2_H_5_OH occurs two times more on [center Si-adatom/Si-rest atom] pair sites compare to [corner Si-adatom/Si-rest atom] pair sites on Si(111)-7 × 7 [see Figure 17 (a)]. Therefore, more Ag atoms are observed on corner Si-adatoms in Figure 20(a), but Ga atoms adsorb selectively on corner Si-adatoms as shown in Figure 20(b), and Zn atoms prefer to adsorb on the site with adjacent Si-adatom by forming a pair as shown in Figure 19(c), which is completely different from the adsorption of Zn by forming Zn_3_ clusters on a clean Si(111)-7 × 7 surface. The results suggest that the metal atoms scout suitable Si-adatom sites on the surface without hopping migration, but the mechanism of selection is not clear.

**Figure 21:**
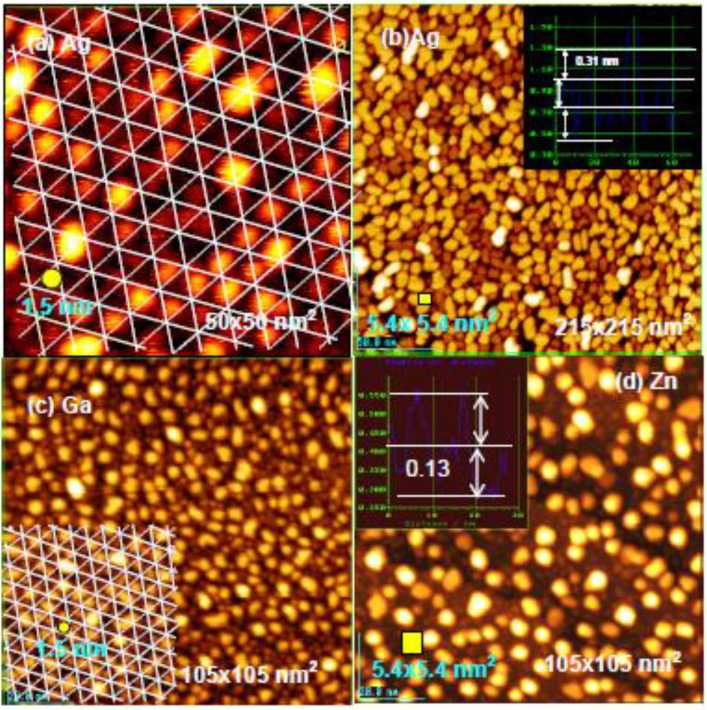
Controlled growth of nano-dots on the Si(111)-7 × 7 C_2_H_5_OH surface; (**a**) Growth of Ag-dots from a sing Ag atoms; (**b**) Growth of Ag-dots into 5 nm crystalline dots. The dots have a similar size as hexagonal mesh (5.4 × 5.4 nm^2^); (**c**) Ga-dots seem to grow in a half unit cells; (**d**) The growth of nano-crystalline Zn-dots may be regulated by a hexagonal mesh. Yellow square indicates the size of 5.4 nm^2^ [71].

If the deposited metal atom cannot migrate by hopping on intact Si-adatoms, surface diffusion of metal atoms may be controlled by their mean free diffusion length on the surface. If this is the case, we could expect the growth of uniform size particles by a uniform diffusion length. As shown in Figure 21(a), rather uniform size Ag-dots (1.5 nm) are formed on the Si(111)-7 × 7-C_2_H_5_OH surface, which may be regulated by a diffusion length of Ag atoms in a half unit cell (ca.1.5 nm, see in Figure 16), that is, the growth of Ag dots is controlled by the kinetics instead of thermodynamics (energy), which suggests a “kinetic controlled molding” for the growth of dots.

When more Ag atoms are deposited, Ag dots grow to a larger size (ca. 5 nm), which is close to the size of hexagonal spacing (5.4 nm) composed of six half unit cells shown in Figure 21(b). Not only the growth of Ag dots but also the growth of Ga and Zn dots seems to be controlled by the kinetics, that is, the size of Ga dots is close to ca1.5 nm and that of Zn dots is close to 5.4 nm, as shown in Figure 21(c) and Figure 21(d) [71].

**Figure 22:**
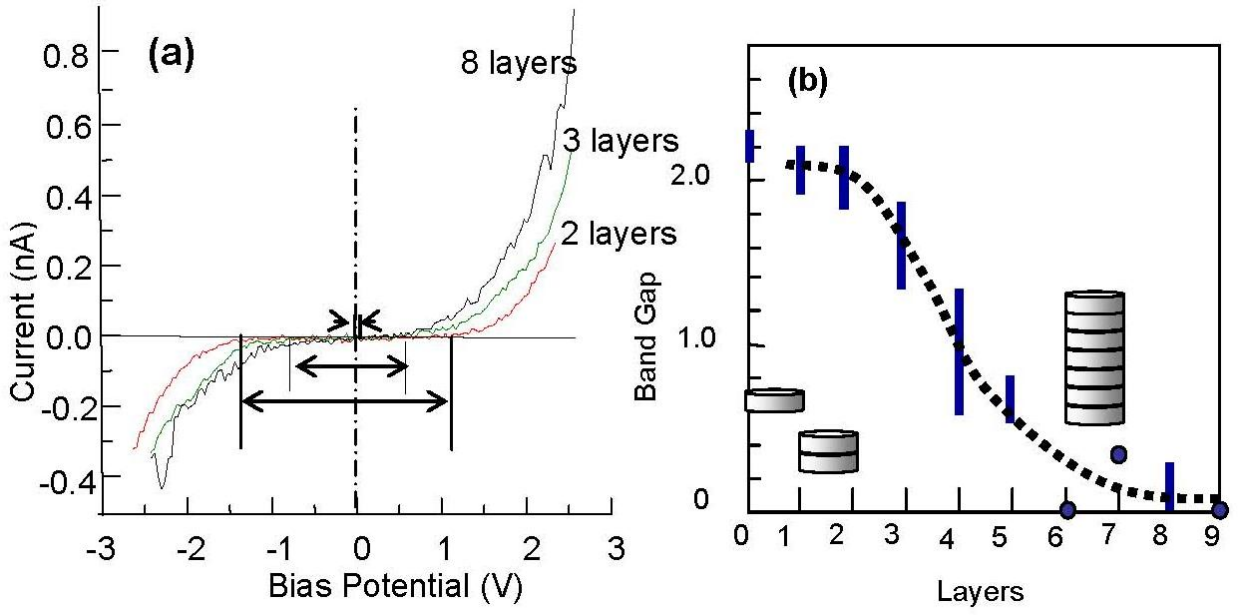
(**a**) STS of Ag dots grown on the Si(111)-7 × 7-C_2_H_5_OH surface; (**b**) Band gap of Ag-dots changes from semiconductor to metal depending on the height [71].

Interestingly, Ag dots seem to grow layer-by-layer by keeping their particle size of ca. 5 nm width on the Si(111)-7 × 7-C_2_H_5_OH surface. The electronic structure of Ag dots depending on the height (layers) was studied by using scanning tunneling spectroscopy. The clean Si(111)-7 × 7 surface is metallic [77], but the Si(111)-7 × 7-C_2_H_5_OH surface has a band gap of 2.2 V [64]. It is known that the atoms or atom cluster of Co, In, Ag, and Sn adsorbed on clean Si(111)-7 × 7 surface are non-metallic [63,70,78,79]. As shown in Figure 22(a), 5 nm width Ag dots having 1–2 layers have a band gap of about 2.0 V, but the band gap of Ag dots becomes narrower when the dot has 3–5 layers and the Ag dots have more than five layers, are metallic as shown in Figure 22(b) [71].

So far, the growth mode of metals or compounds on the surface has been explained by a thermodynamic (energy) relation given by an equation of ΔΓ = Γ_s_ + Γ_f_ − Γ_I_, where Γ_s_ and Γ_f_ are the surface energy of substrate and the developed over-layer, and Γ_I_ is the interfacial energy between them. When the energy relationship becomes Γ_s_ + Γ_f_ > Γ_I_ (ΔΓ > 0), three dimensional growth may take place on the surface. Conversely, when the relation becomes Γ_s_ + Γ_f_ < Γ_I_, (ΔΓ < 0), Stranski-Krastanov type layer-by-layer growth is expected. In this case, the growth mode controlled by the energetic feasibility (thermodynamics) depends on the materials. In contrast, when the growth of materials is controlled by kinetics instead of thermodynamics, a similar mode of growth takes place for different materials as observed on Ag, Ga, and Zn. From this view point, a composite Cu(100) surface made by inlaid square Cu_3_N(100) patches shown in Figure 15 is an interesting surface to study the mechanism of the growth modes of dots or islands, which are controlled by either energetic feasibility or growth kinetics.

**Figure 23:**
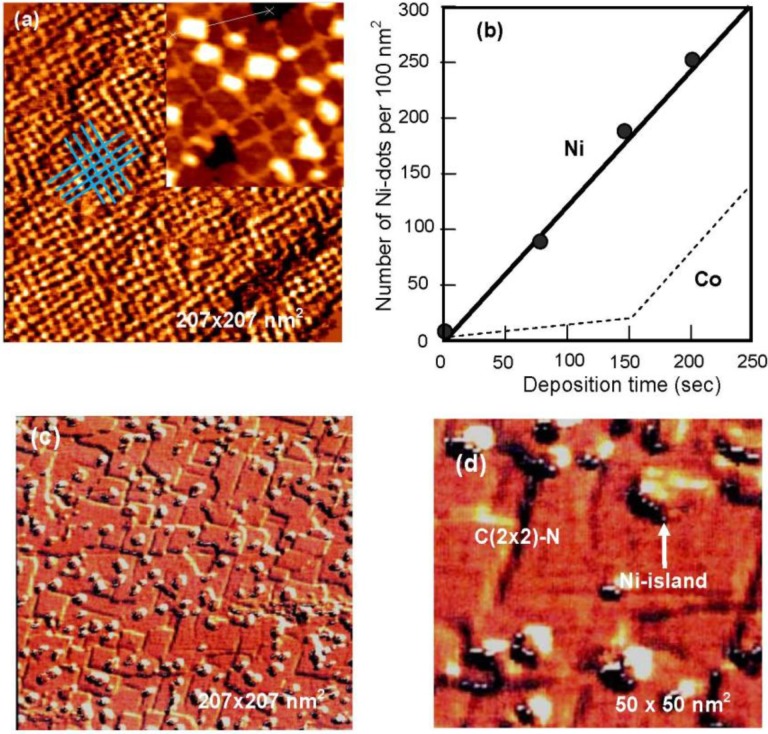
(**a**) Ni-dots formed on a Cu (100) covered with ca. 5 × 5 nm^2^s quare c (2 × 2)-N patches; (**b**) Number of nano-Ni-dots (density) on the surface increase linearly by the deposition time of NI. According to a model shown in Figure 24, Co-dots may increase according to a line shown with broken line; (**c**) Deposited Ni atoms grow randomly by making large Ni-islands when a Cu (100) surface is perfectly covered with Cu_3_N (100) layer as shown in; (**d**) with an expanded image (50 × 50 nm^2^) [81].

A Cu(100) surface covered with ca. 5 nm square c(2 × 2)-N patches has bright lines of clean Cu(100) zones which separate the patches. The line width depends on the nitrogen coverage, that is, the density of c (2 × 2)-N patches because the size of patches is ca. 5 nm square. In fact, the number of square patches (density) on the Cu(100) surface is increased linearly with N-ion bombardment time as shown in Figure 23(b) [81]. Komori *et al.* [82] showed that the brightness of the lines having more than 3 nm width was almost equal to that of the clean Cu(100) surface, but the lines with narrower than 3 nm width look dark, that is, the electronic state of the line Cu(100) zone depends on the width. As it was mentioned above, a square c (2 × 2)-N patch is a Cu_3_N(100) plane inlaid to the Cu(100) surface. Therefore, as the density of square patches increases, the width of the grid-like lines of the clean Cu(100) area becomes narrower and the lattice distortion becomes higher, which may be reflected by the brightness of lines depending on the width. The Cu(100) surface is finally almost covered with Cu_3_N(100) plane as shown in Figure 15(c). An interesting fact is that the Cu_3_N(100) plane is formed not only on the Cu(111) and Cu(110) surfaces but also on the Cu(100) surface by the reaction of Cu atoms with nitrogen ions. It is very similar to the array of Ni_4_C molecule in the Ni(111), Ni(100) and Ni(110) surfaces shown in Figure 14.

When Ni is deposited on the Cu_3_N(100) patches-covered Cu(100) surface, the lattice constant of fcc Ni crystal is 2.5% shorter than that of fcc Cu crystal. On the other hand, Cu-Cu distance in the Cu_3_N(100) plane is ca. 5% longer than that of the Cu crystal. That is, the Ni-layer will have larger lattice mismatch on the Cu_3_N(100) layer compared to that on the Cu(100) surface. On the other hand, a layer-by-layer growth of Ni layer on the clean Cu(100) surface was reported [82], that is, ΔΓ < 0. Taking these facts into account, when Ni is deposited on a square patch Cu(100) surface, Ni atoms may not adsorb on the square c(2 × 2)-N patches but rather on the grid-like Cu(100) area. In fact, when Ni atoms are deposited on a Cu(100) surface covered with square c(2 × 2)-N patches (Cu_3_N(100) plane), growth of Ni nano-dots occurs at the crossings of the grid-like lines of Cu(100) surface area but no dots are formed on the square c(2 × 2)-N patches. On the other hand, when Ni is vaporized on a Cu(100) fully covered with a Cu_3_N(100) layer (c(2 × 2)-N patches) such as shown in Figure 15(c), Ni-islands having two and three atomic layers are randomly formed on the surface even at low Ni coverage, as shown in Figure 23(c), and Figure 23(d) [83], which indicates a Volmer-Wever type growth on the Cu_3_N(100) surface ( ΔΓ > 0). The growth of Ni-dots at the crossing suggests that Ni atoms are provided by diffusing through the grid-like strained Cu(100) narrow lines and are stabilized in less strained crossings by making nano-dots. Dot growth is regulated by the crossing space as shown in Figure 23(a). As a result, the number of uniform sized Ni-dots increases linearly through the origin with respect to the deposition time of Ni as shown in Figure 23(b) [81]. This is a typical example of a kinetic controlled nano-patterning on a composite surface.

The growth of Fe [80,83], and Co [84,85] particles on the Cu(100)-c(2 × 2)-N surface is also controlled by growth kinetics. In the case of Fe, the monolayer islands are first formed at the line crossings of the c(2 × 2)-N square-patches on the Cu(100) surface. With increased deposition of Fe, the Fe monolayer grows along the clean Cu(100) lines, and the Fe-dots in the crossings take double-layered height. It is noteworthy note that the boundary edge of the Fe-layer at the c (2 × 2)-N patches is less strict than that of the Ni-dots. This phenomenon may indicate that the affinity of Fe-layer to square c(2 × 2)-N patches (Cu_3_N(100) plane) may be larger than that of Ni-layer That is, the Fe monolayer bulged out from the clean Cu(100) zone into c(2 × 2)-N patches when the deposition is increased.

**Figure 24:**
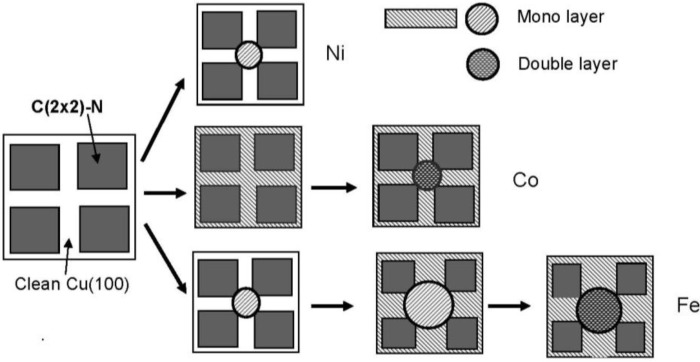
Models of kinetic controlled growth of Ni, Co and Fe nano-dots on the Cu(100) surface covered with c(2 × 2)-N square patches.

In addition, the growth mode of Fe layer on the line or in the crossing is influenced by the evaporation rate of Ni [81], which is also a feature of kinetic controlled molding growth of the dots or layers. On the other hand, deposited Co atoms forms a one-atom layer over the clean Cu(100) lines, and then nano-size of Co dots grow in the crossing with double layer height [see Figure 23 (b)]. As a result, Co-dots are regularly connected with monolayer Co-lines on the Cu(100)-c(2 × 2)-N surface. These three growing modes are illustrated in Figure 24.

Komori *et al.* [80] studied the ferromagnetism of nano-Co particles prepared on a Cu(100)-c(2 × 2)-N surface by using SMOKE at 95 °K. When a Cu(100) is covered with 0.2–0.4 mL of N-atoms, 5 nm c(2 × 2)-N patches are separated with 2 nm width clean Cu (100) lines. Longitudinal magnetic hysteresis of Co film was measured by applying magnetic field along the easy magnetization axis, and obseerved no remanent magnetization of the nano-width Co-film up to an averaged thickness of 1.2 mL, but the film thicker than 1.3 mL had a remanent magnetization. They concluded that the nano-width Co line is similar to the Co film prepared on the Cu(100) surface. They also confirmed that the vanishing temperature of the remanent magnetization depends on the thickness of Co film.

Finally, we could conclude that the growth of nano-materials is controlled by the energetic feasibility as well as by growth kinetics on the designed composite surfaces. The idea proposes “kinetic controlled molding” for preparing nano-size new materials with new properties.

## 6. Conclusions

Self-assembly of atoms, molecules, and quasi-compounds on the surfaces is markedly influenced by weak mutual interactions and the lattice strain induced by the adsorption. Lattice strain influences the self-assembled array of adsorbed species and the formation of nano-composite structures, but the influence is not always on energetic feasibility but on the kinetics so that some nano-structuring is controlled by the kinetics. The driving force for rapid release of metal atoms from the surface in the presence of O_2_ or H_2_ is explained by chemical reactions forming quasi-molecules. Quasi-molecules can migrate rapidly over the surface and undergo self-assembly on the surface by weak interaction. Self-assembled (n × 1) (-Ag-O-) strings on Ag(110), (2 × 1) (-Cu-O-) strings on Cu(110), (3 × 1) and (2 × 1) (-Ni-O-) strings on Ni(110), and (1 × 2) (-Ni-H-) on Ni(110) are typical examples. According to the quasi-compound concept, it may be possible to prepare new composite surfaces by the reaction of quasi-compounds. A typical example is a Ag(110) surface covered with (-Cu-O-) strings prepared by the reaction of (-Ag-O-) stings with Cu-atoms. The (-Cu-O-) strings prepared on the Ag (110) surface undergo self-assembly in a (2 × 2)2mg structure. As the (-Cu-O-) string on the Ag (110) surface is not so stable, it readily decomposes into (Cu_2_)_3_ dots by raising the temperature. Interestingly, a reverse reaction makes (-Cu-O-) strings grow when the (Cu_2_)_3_ dots-covered Ag(110) surface is exposed to O_2_ at room temperature. By forming a nano-composite surface, we could control the growth of nano-materials on it. The growth of nano-metal dots on a composite Cu(100) surface with square patches of Cu_3_N(100) plane as well as that on the Si (111)-7 × 7-C_2_H_5_OH surface would be an example of the “kinetic controlled molding”. The idea of “kinetic controlled molding” will be a valuable concept for the design of new nano-materials.
